# Comparative metabolic study of planktonic and sessile cells in *Salmonella* Enteritidis ATCC 13076: Elucidating metabolic pathways driving biofilm formation

**DOI:** 10.1371/journal.pone.0317420

**Published:** 2025-01-24

**Authors:** Yuliany Guillín, Claudia Ortiz, William Hidalgo

**Affiliations:** 1 Escuela de Biología, Universidad Industrial de Santander, Bucaramanga, Colombia; 2 Escuela de Microbiología y Bioanálisis, Universidad Industrial de Santander, Bucaramanga, Colombia; 3 Escuela de Química, Universidad Industrial de Santander, Bucaramanga, Colombia; Laurentian University, CANADA

## Abstract

Microorganisms tend to accumulate on surfaces, forming aggregates such as biofilms, which grant them resistance to various environmental stressors and antimicrobial agents. This ability has hindered the effective treatment of diseases caused by pathogenic microorganisms, including *Salmonella*, which is responsible for a significant number of deaths worldwide. This study aimed to compare the metabolic profiles of planktonic and sessile cells of *Salmonella* Enteritidis using a metabolomics approach. The metabolites extracted from the bacterial cells were analyzed by LC/MS approach. Raw data were analyzed using Thermo Xcalibur v 3.1 software. For data processing, XCMS was used for feature detection, retention time, correction and alignment. The data matrix was analyzed by uni- and multivariate statistical methods (PCA, PLS-DA, Heatmap) in MetaboAnalyst software v 6.0. A total of 121 metabolites were presumptively identified as differential metabolic characteristics between the two bacterial states, and they were associated with their corresponding metabolic pathways. Among the metabolites that exhibited positive modulation in planktonic cells were proline, phenylalanine, which act as precursors of essential metabolites and part of the stress adaptation mechanisms. In addition, putrescine and cadaverine, play crucial roles in growth, stress response, and cell stability In contrast, the most representative metabolites in sessile cells included lysine, adenosine, purines, pyrimidines, and citrate, mainly associated with maintaining cellular homeostasis, stress response and metabolic regulation. Finally, pathway enrichment analysis identified metabolic changes in 11 pathways, predominantly involving purine and pyrimidine metabolism, arginine and proline metabolism, and vitamin B6 metabolism. These findings facilitated the identification of potential metabolic pathways associated with biofilm formation in the sessile cells of *Salmonella* Enteritidis.

## 1. Introduction

Most bacteria alternate between two modes of microbial growth: a planktonic state, where cells are free-swimming (planktonic), or in a sessile state, where bacteria live attached to inert or organic surfaces, surrounded by a self-produced matrix known as a biofilm [1, 2]. The biofilm matrix contains mainly polysaccharides, proteins, and extracellular DNA [3]. This alternating cycle in which planktonic cells transition to sessile cells within a biofilm, and sessile cells detached from the biofilm to planktonic cells, is mediated by differential gene expression and cellular communication mechanisms such as quorum sensing or cell-cell signaling, in particular with the cyclic secondary messenger di-GMP [4–6]. Biofilm formation occurs in five main stages: (*i*) adhesion of cells to surfaces using flagella, pili, or adhesion proteins [7]; (*ii*) cell monolayer formation, which generates an irreversible adhesion by attachment of bacterial appendages and production of exopolymers [8]; (*iii*) production of the extracellular matrix as a diffusion barrier that prevents the penetration of substances that may affect the integrity of the aggregate [5]; (*iv*) maturation of the biofilm, the microcolonies grow together to form macrocolonies. These macrocolonies then attach to a surface through electrostatic interactions [9] and (*v*) dispersal of the biofilm, which occurs through phenotypic changes in which sessile cells detach from the biofilm and migrate as planktonic cells to colonize new surfaces [2].

The mechanism of biofilm formation enables colonization of a wide range of environments and surfaces and confers to microorganisms an increased resistance to the immune system and conventional drugs, becoming one of the most relevant challenges of current antimicrobial therapy [10–12]. Among several pathogens, *Salmonella enterica*, a Gram-negative bacteria responsible for salmonellosis and a main biofilm-forming microorganism, has been the subject of study due to its resistance and persistence in both host and non-host environments, especially in food processing environments [13, 14]. *Salmonella* is recognized to colonize abiotic surfaces such as plastic, glass, cement, rubber, and stainless steel and biotic surfaces such as plants, epithelial cells, and gallstones [14, 15]. In addition, the World Health Organization has categorized *Salmonella* as a threat of high level due to its resistance to antibiotics such as ciprofloxacin, azithromycin, and ceftriaxone, sometimes needed to treat patients with severe *Salmonella* infections [16].

In this sense, in recent years, research has been focused on understanding the physiology of these microbial communities, as it may be applied in future schemes of diagnostics and treatment [17–20]. Currently, omics tools make it possible to obtain global profiles of microbial physiology, especially metabolomics, which analyses small molecule metabolites in living organisms and is a sensitive and promising tool to study environment-organism interactions [21].

Metabolic changes in these microorganisms become necessary for proper cell growth, maintenance, and function [22, 23]. In contrast with genes and proteins, whose functions are subject to epigenetic regulation and post-translational modifications, metabolites change directly from biochemical activity [22, 24]. Therefore, correlating the dynamic fluxes of metabolites in the cells with metabolic activity provides insight into the phenotype and its associated changes due to cellular response to generic or environmental stimuli [22, 24, 25]. Here, it was compared the metabolic profiles of planktonic and sessile cells of *Salmonella* to explore the main biosynthetic pathways involved in both state of this microorganism.

## 2. Materials and methods

### 2.1 Bacterial strain and growth conditions

*Salmonella enterica* serovar Enteritidis ATCC 13076 strain was purchased from the American Type Culture Collection (ATCC; Rockville, MD, USA). It was cultured in M63 medium and incubated overnight at 37°C to stationary phase growth, and then used for subsequent planktonic and biofilm studies [26]. All reactions and biofilm assays were carried out using Milli-Q water of 18.2 Ω resistivity, from the Smart 2 Pure instrument (Thermo Fisher Scientific, Waltham, MA, USA).

### 2.2 Planktonic culture growth

Planktonic cultures were prepared by inoculating 18 mL of M63 medium with 2 mL of the overnight culture. The culture was adjusted to reach a concentration of ~5 × 10^6^ CFU/mL and added to wells of a 96-well microplate. The plates were then incubated for 3 h at 37°C with shaking (200 rpm). After this time, bacterial cultures were then washed three times with 0.1% (*w/v*) peptone solution and centrifuged at 4000 × g for 10 min at 4°C (IEC CL31R Multispeed centrifuge, Thermo scientific, Waltham, MA, USA with rotor Thermo Scientific 75003424 Fixed Angle 24 x 1.5/2.0). Metabolite extraction from the planktonic cells was performed during the third hour of microbial growth kinetics, which occurs in the exponential phase [27].

### 2.3 Biofilm culture growth

Biofilm formation was performed using microplates that contained glass coupons measuring 1 × 1.5 cm. An inoculum of ~1 × 10^6^ CFU/mL was added to a microplate and incubated for 24 h at 37°C without shaking. Then, glass coupons were washed three times with 0.1% (*w/v*) peptone solution to eliminate free-floating planktonic bacteria. The biofilm-associated cells were then collected using sterile spatulas for subsequent metabolite extraction. Furthermore, bacterial cultures were then washed three times with 0.1% (*w/v*) peptone solution and centrifuged at 4000 × g for 10 min at 4°C [27].

### 2.4 Metabolomic analysis

#### 2.4.1 Metabolite extraction

The intracellular metabolites were extracted as described in previous publication by Zhou *et al*. [27] with minor modification. Briefly, cell samples were homogenized with 500 μL of 50% *(v/v)* cold methanol (MeOH aq.) mixed with 500 μL of 50% (*v/v*) acetonitrile (ACN aq) and subjected to sonication using 130-Watt ultrasonic processor (Cole–Parmer Instruments, Vernon Hills, IL, USA) (ON time = 10, OFF time = 45, and amplitude = 80%) and centrifuged at 13000 g for 20 min at 4°C. Supernatant of each sample was collected and concentrated in a Savant Speed Vac SPD120 vacuum concentrator (Thermo Fisher Scientific, Waltham, MA, USA). The dried extracts were resuspended with 400 μL of 70% (*v/v*) methanol solution containing the internal standards Carbobenzoxyglycyl-L-tyrosin and caffeine (99.9% purity) at 5 μM. Quality control (QC) samples were prepared as described in previous studies [28–30]. All experiments were carried out with nine biological replicates (n = 9).

#### 2.4.2 Data acquisition and analysis by UHPLC-ESI-Orbitrap/HRMS

Each methanolic extract was analyzed using a UHPLC with a Dionex Ultimate 3000 connected to an OrbitrapTM Exactive mass spectrometer and equipped with an ESI source (Thermo Fisher Scientific, Sunnyvale, CA, USA). Chromatographic and mass spectrometry parameters were implemented as described in previous work [31].

#### 2.4.3 Data preprocessing

Raw data file was analyzed using Thermo Xcalibur v 3.1 software (Thermo Scientific, Sunnyvale, CA, USA), additionally converted to mzXML format using the MSConvert file conversion tool (ProteoWizard 3.0, accessed on February 10, 2024). For data processing, XCMS online platform was used to feature detection, retention time correction and alignment [32]. Parameter setting for LC data, centWave method was used for the selection of peaks mode for feature detection (Δppm = 5 ppm, minimum peak width = 5 s, and maximum peak width = 20 s); Correction of the retention time was performed with an obiwarp method (profStep = 1) and, for chromatogram alignment, minfrac = 5, bw = 5, mzwid = 0.025. Afterward, the features (*m/*z) from the matrix data of Quality Control (QC) samples with a coefficient of variation (CV) >25% were removed for the analysis.

#### 2.4.4 Data analysis

To determine the differences in the metabolic profiles between planktonic and biofilm cells, uni- and multivariate analysis was performed. Statistical analysis was performed with MetaboAnalyst software version 6.0 (https://www.metaboanalyst.ca/) (accessed February 10, 2024) [33]. All data were normalized by sum and log transformed (base 10) both positive and negative ion acquisition mode; additionally, data acquired for negative ion mode were scaled by autoscaling (centered on the mean and divided by the standard deviation of each variable). The data matrix was a tabular representation in which the rows correspond to the features (*m/z* values) detected in the samples, and the columns represent the different samples analyzed. Each value in the matrix contained the relative intensity of a specific feature in a sample. Multivariate statistical analysis included principal component analysis (PCA) and partial least squares discriminant analysis (PLS-DA), which was used to discriminate sample patterns. Mass/charge ratios (*m/z*) that contributed significantly to clustering and discrimination were identified according to the variable of importance in the projection (VIP) and a false discovery rate (FDR) <0.05 (to determine if changes in their levels are statistically significant between conditions). Additionally, a heatmap was performed to determine patterns or groups (cluster) amount the samples, along with the fold change (FC) analysis to obtain a relative quantification of the metabolites, whose values from 0–0.5 correspond to metabolites that presented a decrease in their abundance, and values greater than 2 indicate metabolites with an increase in their abundance. Furthermore, a t-test was performed to determine the statistical variation of the measurements of the two groups (sessile and planktonic cells) and a volcano plot, which allows for the statistical significance (*p* value) to be related to the magnitude of the change (FC) and Log2(FC) to transforms the ratio into a symmetric scale that simplifies interpretation [34, 35]. Finally, volcano plot analysis was performed to allowed detection of the differential features (*m/*z) by Student’s t-test with the *p* value less than 0.05 and fold-change (FC) more than 2 or less than 0.5. These features were presumptively identified through CEU Mass Mediator software version 3.0 (http://ceumass.eps.uspceu.es/) (accessed on February 25, 2024) [36] and by searching mass spectrum form libraries such as Mass Bank (https://massbank.eu/MassBank/Search) (accessed on February 25, 2024) and the Human Metabolome Database (https://hmdb.ca/) (accessed on February 25, 2024). Selected critical pathways were further checked in the pathway library on The Kyoto Encyclopedia of Genes and Genomes pathway database (https://www.genome.jp/kegg/) (accessed on February 26, 2024).

## 3. Results and discussion

### 3.1. Metabolome analysis

**Fig 1:**
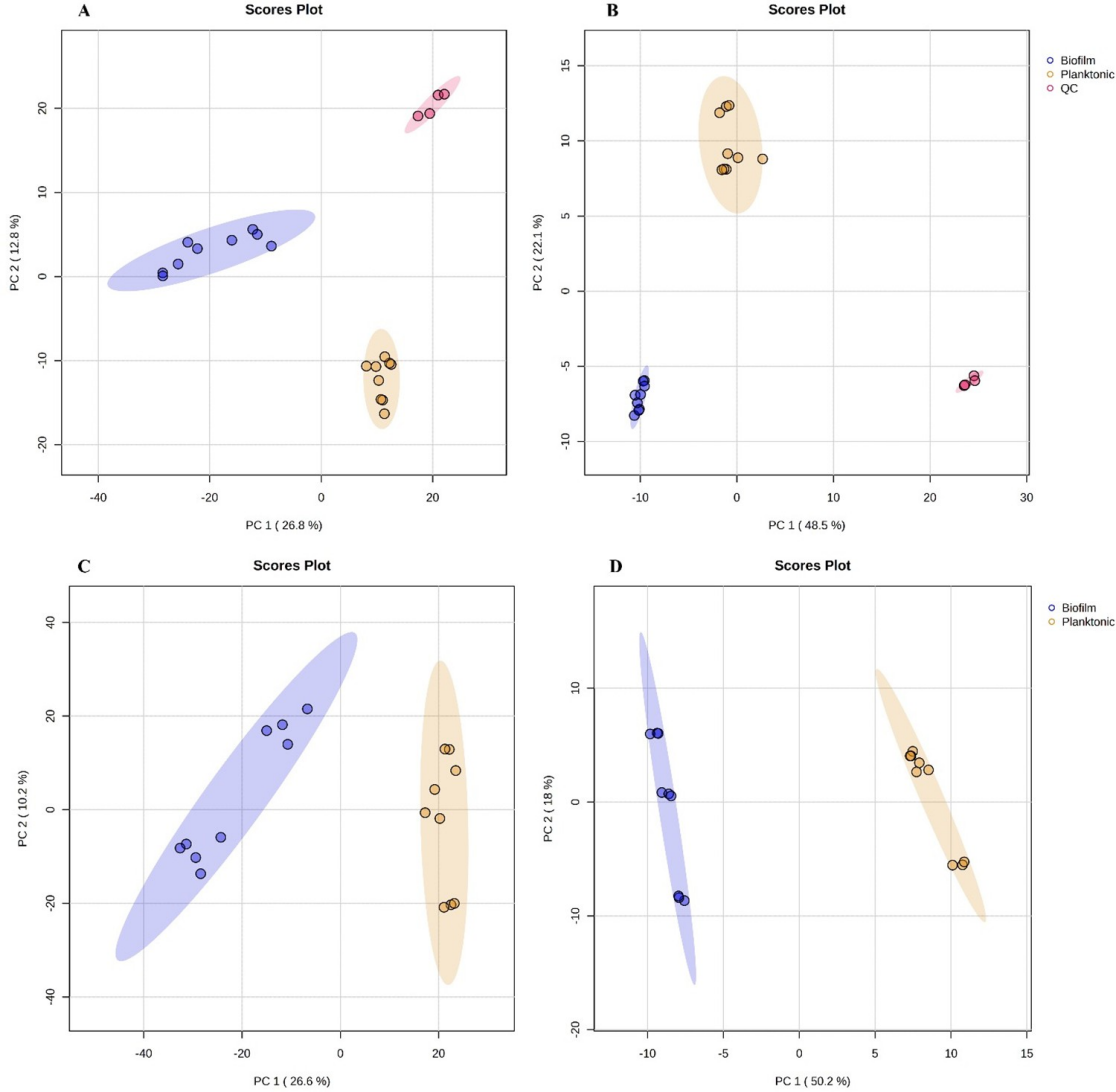
Scoring plot representing PCA results based on UHPLC-ESI-Orbitrap/HRMS data matrix on planktonic and sessile cells of *S*. Enteritidis. (A) PCA including QC samples with data obtained from negative ion acquisition mode. (B) PCA including QC samples with data from positive ion acquisition mode. (C) PCA with data from negative ion acquisition mode. (D) PCA with data from positive ion acquisition mode. Biofilm (blue color), Planktonic (yellow color) and QC (red color).

Untargeted metabolomics analysis was performed on planktonic and sessile cells to identify the differences in their metabolic profiles. The raw data matrix contained 3378 and 5864 features for the negative (NI) and positive ion (PI) acquisition modes, respectively. After filtering with quality control samples (CV >25%), a matrix with 1971 and 3481 features for the NI and PI acquisition modes, respectively, was obtained (S1 and S2 Tables in S1 File). This matrix was then analyzed using uni- and multivariate statistical methods. Initially, a principal component analysis (PCA) was performed, which showed a well-defined separation of the planktonic cells (planktonic), sessile cells (biofilm), and quality control samples (QC) by both acquisition modes (Fig 1A and 1B), indicating that the observed differences were linked to biological variations [37]. Furthermore, PCA plots were generated for the planktonic and sessile cells, the results revealed a separation between both groups by both ionization modes (Fig 1C and 1D). The two principal components (PC1 and PC2) explained 36.8% (PC1: 26.6% and PC2: 10.2%) and 68.2% (PC1: 50.2% and PC2:18%) of the total variability in the data sets for the NI and PI acquisition modes, respectively.

**Fig 2:**
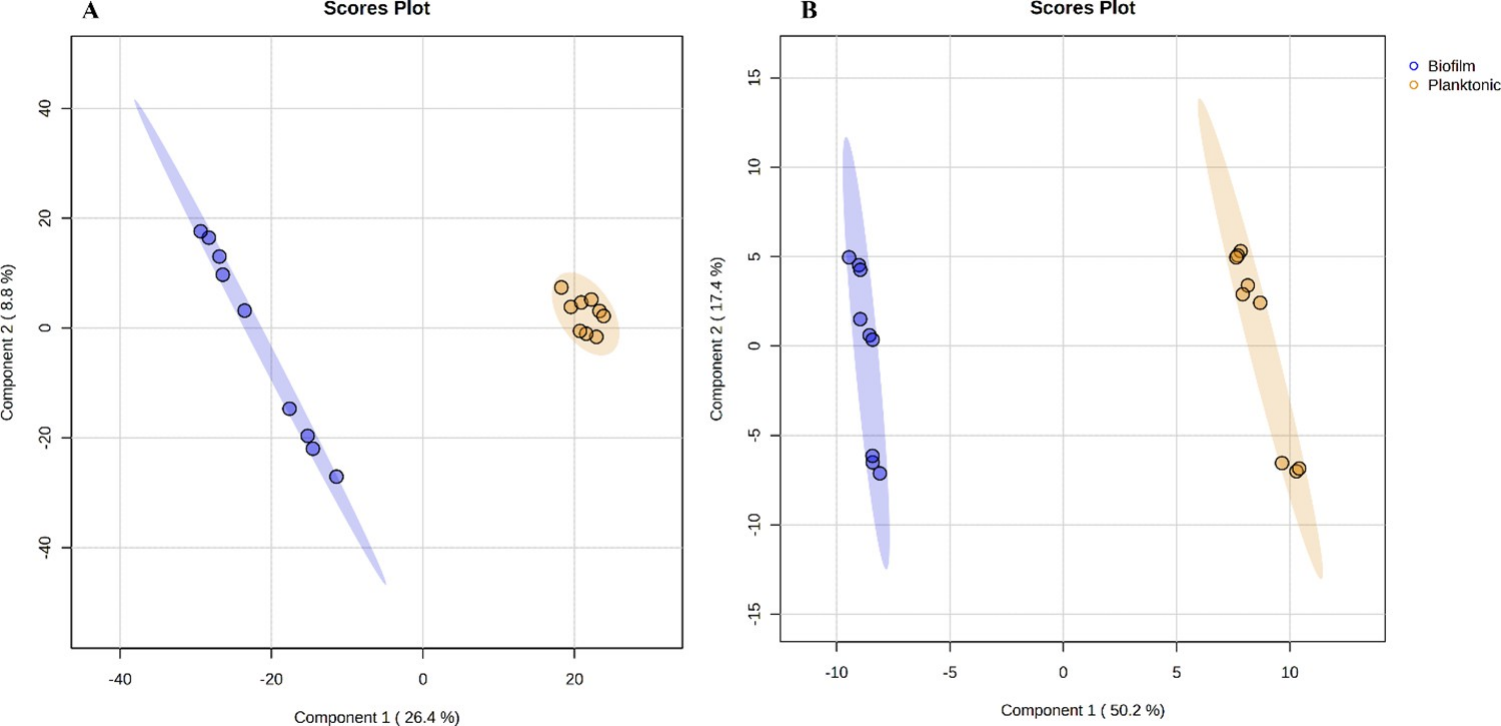
Scoring plot representing PLS-DA results based on UHPLC-ESI-Orbitrap/HRMS data matrix on planktonic cells and sessile cells of *S*. Enteritidis. (A) PLS-DA with data from negative ion acquisition mode. (B) PLS-DA with data from positive ion acquisition mode. Biofilm (blue color), Planktonic (yellow color).

Then, to maximize the separation and identify changes between both samples, we conducted a supervised partial least-squares-discriminant analysis (PLS-DA) as shown in Fig 2A and 2B. The R2 and Q2 parameters indicated a model with high predictability and reliability (0.95 and 0.89) for the NI mode and 0.99 and 0.98 for the PI mode, respectively. Overall, the results demonstrated differences in the metabolic profiles of planktonic and sessile cells of *Salmonella*, which are similar to those reported for bacteria such as *Brucella abortus*, *Escherichia coli*, *Lactobacillus paraplantarum*, *Bifidobacterium bifidum* [28, 38–40].

To clearly distinguish the different metabolites of these two groups, those displaying significant changes (P < 0.05, VIP > 1, and fold change Log2FC) were selected and identified by a comparison of the mass spectrum, fragment ions, and exact mass with those reported in the literature and databases. In total, 121 metabolites were putatively identified (S3 Table in S1 File, level 2 of metabolic identification, according to the Metabolomics Standards Initiative), and 57 and 64 significant metabolites were identified for the (PI) and (NI) acquisition modes, respectively. Of the metabolites identified for the NI mode, 15 were positively modulated and 42 were negatively modulated. The identified metabolites included adenine, malic acid, guanosine, uridine, guanine, isoleucine, phenylalanine, and citric acid. For the PI mode, 8 metabolites were positively modulated while 56 were negatively modulated and metabolites such as glutathione, inosine, cadaverine, putrescine, proline, lysine, alanine, tyrosine, among others, were identified.

**Fig 3:**
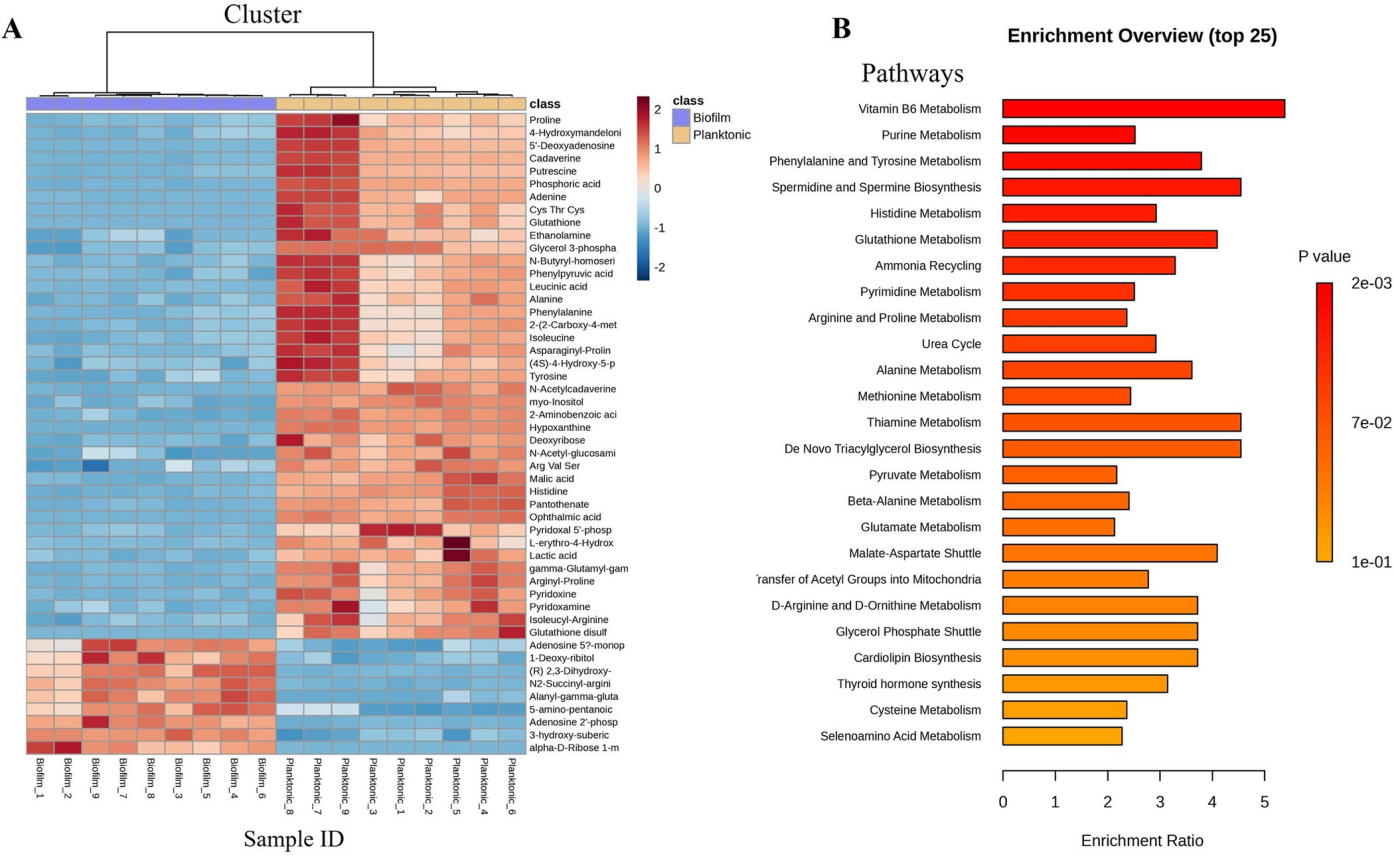
General metabolic profile of planktonic cells and sessile cells of *Salmonella* Enteritidis. (A) Heat map showing metabolites representative of the metabolomic profile of *Salmonella* biofilm and planktonic cells. Data are displayed by nonaplicate as a colored map reflecting the relative abundances of each metabolite identified as characteristic for the groups of samples (red area indicates an increase in metabolite abundance, and blue area a decrease). (B) Enrichment analysis of metabolic pathways based on significantly different metabolites.

The heatmap analysis facilitated the interpretation of chemical abundance data in both planktonic and sessile cells (clusters) (Fig 3A), allowing the identification of variations in their metabolic profiles. Two subclusters were identified: the first encompasses most metabolites whose abundance is negatively regulated in sessile cells, suggesting differences in the metabolism of amino acids such as proline, alanine, and histidine, as well as polyamines, nucleotides like adenine, and metabolites involved in central carbon metabolism. These variations may be associated with reduced metabolic rates in sessile cells. This finding is consistent with several studies, in which biofilms formed by *Pseudomonas aeruginosa*, *Clostridium perfringes*, and *Bifidobacterium bifidum*, show less active metabolic activity in sessile cells as compared to their counterpart planktonic cells, associated with the low abundance of nutrients inside many gradients of biofilm [28, 41, 42].

The second subcluster organizes metabolites that are positively modulated. In this instance, a smaller number of metabolites were identified, e.g., adenosine, the positive induction of this metabolite may be linked to an increase in both AMP and adenosine, which are associated with the metabolic profiles of mature cells that primarily aim to sustain cellular energy through ATP synthesis and serve as signaling molecules [43]. Additionally, 1-deoxyribitol may act as a crucial precursor in the synthesis of matrix components, thereby aiding in the development and stability of biofilms [44, 45]. Moreover, alpha-D-Ribose 1-methylphosphonate 5-triphosphate has a multifaceted role in biofilm formation due to its involvement in phosphonate metabolism, nucleotide biosynthesis and, in addition its presence within microbial communities can enhance the resistance and structural integrity of biofilms [46, 47]. These findings indicate that the metabolites identified may play significant roles in the regulatory, communicative, and maintenance functions of the *Salmonella* biofilm.

Additionally, depending on the stage of biofilm, some metabolites are modulated positively or negatively. Recent studies have reported that during the early stages, an increase in pentoses and amino acids occurs (8–16 h), while in the intermediate stages, a decrease in glycolysis metabolites and amino acids is observed (16–20 h). Finally, in the mature stages of the biofilm an increase in citric acid intermediates and nucleotide synthesis (20 to 24 h) [48], according to this panorama we could mention that the metabolic characteristics of the *Salmonella* biofilm at 24 h are similar to those of a mature biofilm due to an increase in metabolites such as citrate, 2,5-dioxopentanoate, nucleotides and nucleosides such as AMP and adenosine, respectively, and a decrease in the metabolism of some amino acids such as histidine and proline, required during the early stages of biofilm formation.

**Fig 4:**
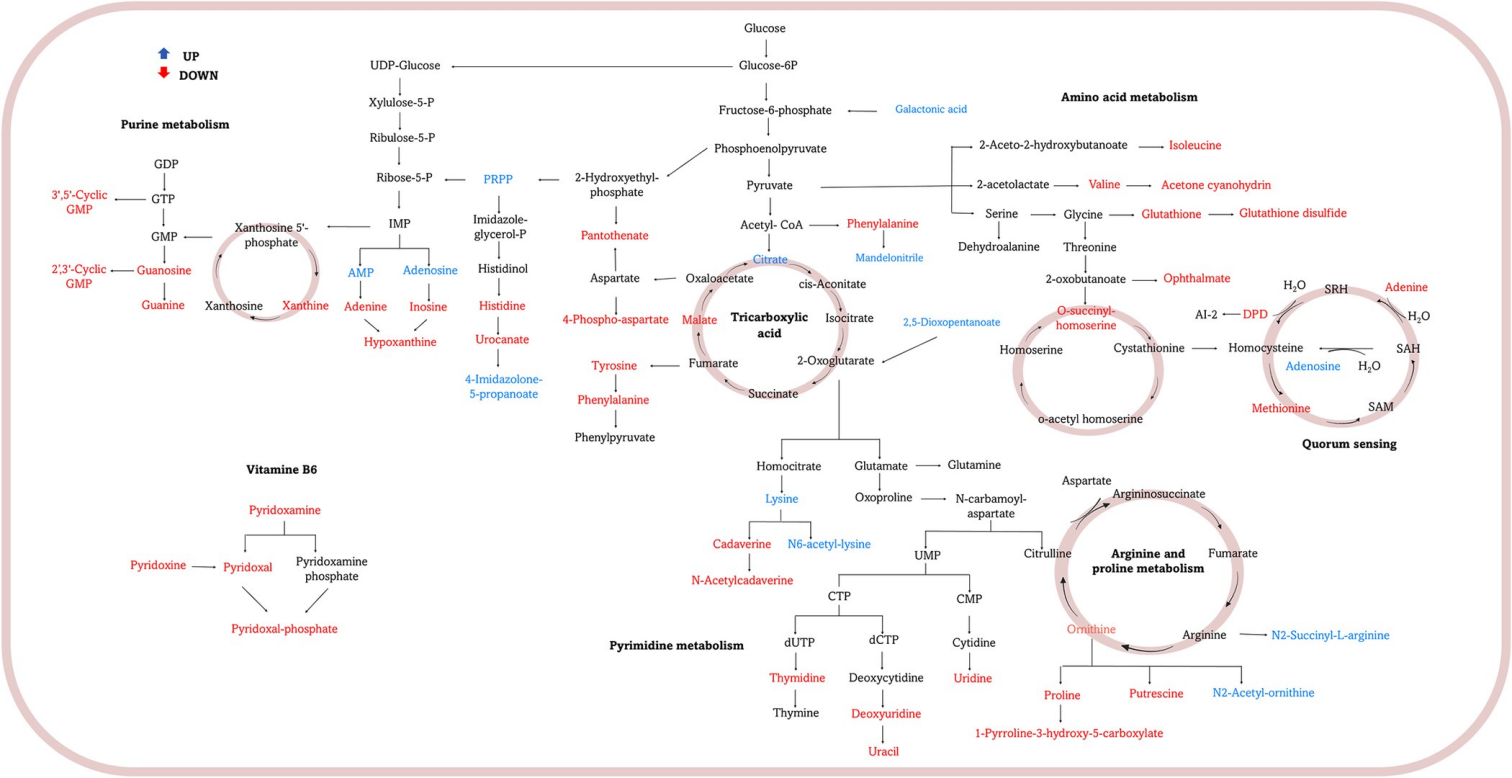
Metabolite changes between pathways in *Salmonella* cells in biofilm and planktonic states. Compared to planktonic cells, the increased metabolites in biofilm cells are represented in blue, while the decreased metabolites are shown in red.

Subsequently, the pathways associated with the metabolic profiles of planktonic cells and sessile cells were identified through enrichment analysis in MetaboAnalyst. The results showed that the main differential pathways were vitamin B6 metabolism, purine, and pyrimidine metabolism, metabolism of amino acids such as phenylalanine, tyrosine, histidine, methionine, arginine, and proline, and glutathione metabolism, among others (Fig 3B) The metabolites detected for each of these pathways were mapped and represented in Fig 4.

The metabolites associated with the vitamin B6 pathways were pyridoxine (PN), pyridoxamine (PM), and pyridoxal phosphate (PLP), which were differentially modulated in planktonic and sessile cells. These vitamers act in a wide range of biochemical reactions, whose function is related to the regulation of basic cellular metabolism and overall physiology, maintaining cellular homeostasis [49]. PLP is the most important of the vitamers, acting as a cofactor for numerous enzymes, including racemases, decarboxylases and transaminases, making this cofactor essential for cellular function. In addition, it is related to amino acid biosynthesis, therefore, an increase in PLP is associated with an increase in some amino acids, such as methionine and proline [50]. This is consistent with our results since these amino acids were positively modulated in planktonic cells, indicating that they are essential in the metabolism of these cells. On the other hand, in sessile cells they remain negatively modulated and increase when subjected to stresses such as the effects generated by some antimicrobials, where they were found to be positively modulated, possibly to contribute to the regulation of homeostasis [30].

However, a similar panorama occurs in the metabolism of purines and pyrimidines where guanine, guanosine, adenine, xanthine, inosine, GMP, thymidine, uracil, and uridine were positively modulated in planktonic cells. Nucleotide metabolism is essential for all organisms, as they are not only of the scaffolding of DNA and RNA, but also act as constituents of many coenzymes involved in energy transport reactions, transfer of organic molecules, and oxidation-reduction reactions [51]. In the case of biofilm cells, these have been identified as components of the early stages of biofilm formation, where they are related to the synthesis of extracellular DNA present in the exopolysaccharide matrix and in the regulation of curli proteins and cellulase fibers, important features in the adhesion stages [52, 53]. However, as mentioned above, depending on the stage of biofilm formation, the metabolic profile will change according to the requirements of each stage. The long-term tendency for both purine and pyrimidine biosynthesis in sessile cells showed negative modulation, which coincides with the generalized decrease in nucleotide and deoxynucleotide levels, similar findings found in late-developing *Bacillus subtilis* biofilms [48]. Disruption of pyrimidine biosynthesis can act as a signal for severe nutrient starvation, which can in turn prevent biofilm formation and promote biofilm dispersal [52].

Nevertheless, metabolites such as Phosphoribosyl pyrophosphate (PRPP), involved in the synthesis of purines and pyrimidines, showed a positive modulation. A positive induction of this metabolite could be related with an increment of AMP and adenosine, which are associated with metabolic profiles of mature cells, whose principal function is to maintain cellular energy through ATP synthesis and act as signaling molecules [43]. Other metabolites that could be contributing to maintaining the energetic metabolism of the biofilm include galactic acid which can enter glycolysis through the fructose-6-P pathway. Additionally, in the central carbon cycle also was observed an increase in metabolites such as citrate, and the precursor of 2-oxoglutarate, 2,5-dioxopentanoate; whereas malate was negatively modulated. Citrate, a crucial intermediate in the tricarboxylic acid (TCA) cycle, has been reported to play a significant role in biofilm development in microorganisms. For instance, in Candida albicans, the citrate cycle has been identified as one of the key pathways enriched during mature biofilm formation [54]. Similarly, Pisithkul *et al*. [55] demonstrated that the accumulation of TCA cycle intermediates, such as citrate, is linked to enhanced biofilm formation in *Bacillus subtilis*. This metabolic remodeling is essential for the transition from a planktonic to a biofilm state, highlighting the importance of citrate in energy metabolism and biofilm maturation [56].

Amino acid metabolism plays a crucial role in efficient energy utilization, maintenance of a balanced redox state, and adaptation to various environmental conditions. The results provided evidence of the metabolic requirements for planktonic and sessile cells. In the metabolic profile of planktonic cells, the positively modulated amino acids were: alanine, an important amino acid for wall synthesis; methionine, a proteinogenic amino acid of vital importance for initiating protein synthesis and contributing to maintaining the balance of reactive oxygen species; proline serves as osmotic regulator, and is closely linked to the cellular response to environmental stressors. Additionally, the metabolic pathways involving proline can affect the overall energy balance, as proline can be converted into other metabolites that are essential for energy production and cellular maintenance [57]. Planktonic cells typically exhibit a more optimized metabolism, which they use for rapid growth and adaptation to change environmental conditions [58]. In contrast, sessile cells may utilize proline in a different manner, incorporating it into structural components or using it to enhance biofilm resilience against antimicrobial agents [59]; phenylalanine is an essential amino acid in planktonic cells, playing a pivotal role in energy metabolism, protein synthesis, and the production of secondary metabolites. Tyrosine, valine, and isoleucine, are essential components of proteins in planktonic cells; and also they are involved in biological processes like metabolic regulation, signaling, and intercellular communication. Their functions are diverse and significantly affect both the physiological state of the cells and their interactions with the environment [60], among others.

On the other hand, the metabolic profile of sessile cells was found to have an increase in amino acids like lysine, as well as acetylated amino acids like N6-acetyl-lysine and N2-acetyl-ornithine. These compounds probably are involved as energy providers for the biofilm formation, stability, and susceptibility to antimicrobial agents This metabolic profile was similar to that found by Sadiq *et al*., [28] and Leggett *et al*., [61], where a lower abundance of amino acids were characteristic in sessile cells, and correlated with less energy production and a decrease in metabolic activities during 24 h biofilm for *Bifidubacterirum* and 48 h for *Pseudomonas*, respectively, which could indicate that the biofilm had already reached maturity. Similarly, Wong *et al*., [62], revealed that in the mature biofilm of *Salmonella enterica* serovar Typhimurium, the amount of amino acids showed a downward trend. Therefore, amino acid metabolism at the cellular level could reflect the maturation state of the biofilm in *Salmonella*.

Urea metabolism also showed notable differences between planktonic and sessile cells. Urea, produced during amino acid decarboxylation, provides further evidence of the importance of amino acids in survival. Several intermediates of the pathway were found to be significantly increased in planktonic cells and decreased in *Salmonella* sessile cells, indicating that they deplete in response to the rapid flux through this system, which is consistent with the results obtained by Stipetic *et al*., [63] where the same metabolic profile was evidenced in *Staphylococcus* biofilms.

Finally, some specific quorum sensing molecules, which is one of the cellular communication mechanisms involved in biofilm formation, were positively modulated in planktonic cells, among them 4-Hydroxy-5-phosphooxypentane-2,3-dione (DPD), which acts as a precursor of autoinducer-2 (AI-2), should stimulate biofilm formation and alters its architecture by stimulating flagellar motion and motility [64]. Sadiq *et al*., [53] found that some genes related to quorum sensing were more highly expressed in planktonic cells of biofilm-forming microorganisms, possibly because these molecules are indispensable in the initial stages of biofilm formation. Additionally, methionine was positively modulated in planktonic cells, this amino acid not only determines the biosynthesis of S-Adenosyl methionine (SAM), which, in turn, influences the synthesis of Quorum sensing (QS) molecules, but also has a close relationship to other functional amino acids, especially glycine and serine. These amino acids are important functional metabolites in the one-carbon pathway that influence nucleic acid, vitamins, and other physiological activities [65].

## 4. Conclusions

Analysis of metabolic profiles revealed differences between planktonic and sessile *Salmonella* Enteritidis. The main differences included a decreased metabolic activity in sessile cells after 24 h of biofilm formation, whereas planktonic cells showed a positive modulation of most metabolites. Although no definitive biomarker was identified to differentiate between planktonic and sessile cells, several candidates may warrant further investigation, such as those involved in biofilm-specific metabolic pathways, quorum sensing, or extracellular matrix production., this analysis offers novel and pioneering insights into the chemical profiles associated with each stage and their metabolic pathways. This information could be valuable in the quest for potential therapeutic targets for the development of new compounds or natural products with anti-biofilm activity against *Salmonella* strains.

## Supporting information

S1 File This file contains three tables summarizing metabolomics data for *Salmonella* Enteritidis ATCC 13076. S1 Table includes features detected in negative ion mode for planktonic and sessile cells. S2 Table includes features detected in positive ion mode. S3 Table lists presumptively identified metabolites and their modulation between sessile and planktonic cells.
(ZIP)
